# Randomised Trial Shows Readymade Oral Nutritional Supplements in Older Malnourished People in the Community Improve Total Nutrient Intakes and Meet More Dietary Reference Values Without Reducing Intake from the Diet

**DOI:** 10.3390/nu17152474

**Published:** 2025-07-29

**Authors:** Marinos Elia, Trevor R. Smith, Abbie L. Cawood, Emily R. Walters, Rebecca J. Stratton

**Affiliations:** 1 Human Development and Health, Faculty of Medicine, MP113, Southampton General Hospital, University of Southampton, Southampton SO16 6YD, UK; elia@soton.ac.uk (M.E.); a.l.cawood@soton.ac.uk (A.L.C.); 2 Department of Gastroenterology, University Hospitals Southampton NHS Foundation Trust, Southampton General Hospital, Southampton, SO16 6YD, UK; trevor.smith@uhs.nhs.uk (T.R.S.); brightnutrition@icloud.com (E.R.W.); 3 Research & Innovation, Danone Nutricia Global Research Centre, 3584 CT Utrecht, The Netherlands

**Keywords:** malnutrition, dietary reference values, supplementation, oral nutritional supplements, community, primary care, energy, micronutrients, displacement

## Abstract

**Background:** There is little information about the effectiveness of oral nutritional supplements (ONS) in combatting nutrient inadequacies in primary care, where most malnutrition exists. **Aim:** To examine the extent to which readymade ONS add or displace the nutrients consumed in the diet and their impact on combatting dietary inadequacies. **Methods:** 308 free-living people >50 years with medium + high risk of malnutrition (Malnutrition Universal Screening Tool) were randomised to receive readymade low volume (2.4 kcal/mL), liquid ONS plus dietary advice (ONS + DA) or dietary advice alone (DA). Intake was assessed at baseline (24 h recall) and 4-weekly for 12 weeks (3-day diet record). Total nutrient intake was benchmarked against UK and European dietary reference values (DRVs). The proportion of energy and nutrients from the ONS that added or displaced those from the diet (net addition/displacement) was calculated. **Results:** ONS + DA led to significantly greater total energy and nutritional intakes, with 25/29 nutrient intakes significantly higher than with DA alone. There were no significant differences in dietary energy and nutrient intakes from food between the groups. There was little or no displacement of nutrients from the diet, with over 90% of the energy and nutrients consumed in the ONS additive to the diet. ONS + DA more than halved the number of people with nutrient intakes that failed to meet DRVs and the number of nutrients per person that did not meet DRVs compared to DA alone. **Conclusions:** Supplementation with readymade, low volume (2.4 kcal/mL) liquid ONS overcomes most nutrient intake inadequacies in malnourished older people in primary care without significantly reducing intake from the diet. This makes ONS an effective way to improve nutritional intakes above dietary advice alone to improve the outcomes for the management of older people at risk of malnutrition.

## 1. Introduction

The effectiveness of oral nutritional supplements (ONS) in improving functional, clinical, and economic outcomes in malnourished people has been repeatedly reported in systematic reviews and meta-analyses [1,2,3,4,5]. Much emphasis has been given to their role in improving total energy and protein intakes more than food-only and dietary advice (DA) approaches. However, the impact of multi-nutrient ONS on the intake of other dietary constituents is also important and less explored [6], especially in older people with disease-related malnutrition, where intakes of a range of vitamins and minerals may be lower than required and deficiencies prevalent [6,7,8]. For example, inadequate provision of potassium and phosphate can prevent the accretion [6] and function of lean tissue. Furthermore, deficiency of many vitamins and trace elements can reduce appetite and predispose to more severe or additional deficiencies [6]. Therefore, compliance or adherence to the nutritional prescription, including ONS, is important [5,9,10,11]. However, to maximally improve total nutrient intake, over and above that of food alone, ideally ONS should have a minimal effect on appetite so that food intake is not reduced, or even a stimulatory effect on appetite, leading to improvements in both dietary and total nutrient intake [5,9,10,11].

Indeed, ONS are typically used alongside the diet and a key issue is the extent to which ONS impacts the nutrient intake from the diet i.e., the extent to which energy and nutrients consumed in the ONS add to those taken from food (improving total nutrient intake) or displace them (so there is less/no improvement in total nutrient intake). The effectiveness of ONS at improving outcomes depends on their ability to significantly improve nutrient intake [6,7,8]. If there is a compensatory reduction in the diet (e.g., appetite is reduced by ONS and the ONS displaces food intake), then improvements in outcomes are less likely or will be attenuated. In contrast, outcomes are more likely to improve or be enhanced when there is little or no displacement of the diet with ONS (e.g., no suppression of appetite, or even appetite stimulation, with the nutrients in the ONS adding to what is eaten in the diet).

An analysis of 24 studies outside hospitals found that, on average, only 31% of ONS energy displaced that from food, which meant that the majority (69%) of the ONS energy consumed added to dietary intake [6]. A greater proportion of ONS energy was found to add to dietary intake in studies involving people with a mean body mass index under 20 kg/m^2^ than over 20 kg/m^2^. However, the variation between studies was large, ranging from 0% (no net benefit in net energy provision) to over 100% of ONS energy adding to dietary intake (implying stimulation of food intake with the use of ONS). These results should be interpreted with caution since not all data were robust, some of the sample sizes were small, and different methods were used to calculate energy compensation in subjects with variable nutritional status. Nevertheless, the potential clinical implications of ONS use displacing energy and nutrients from the diet are enormous. However, it is remarkable that this area of investigation has progressed little and has not been extended to explore the intake of a wide range of nutrients, including minerals and vitamins that contribute to patient recovery.

There is also remarkably little information on the effects of ONS or dietary advice on the total intake of vitamins, trace elements and other nutrients in people in the community setting. Although a handful of studies have reported the impact of ONS on the intake of a range of nutrients in institutionalised elderly subjects [12,13,14,15], in patients receiving chemo/radiotherapy in hospital for head and neck cancer [16], and in those discharged from hospital with cardiopulmonary problems [17], there are fewer data in the community, where most malnutrition exists and ONS prescribed.

Therefore, this study examined in detail the dietary and ONS nutrient intake data obtained in a large, randomised trial of supplementation with ONS (in addition to dietary advice) versus dietary advice alone in general practice. The clinical benefits of the supplementation included a reduction in healthcare use, specifically a reduction in healthcare professional home visits by 34%, emergency admissions by 50%, and length of hospitalisation by 62% [18].

The present study aimed to examine one of the secondary outcomes of the trial (nutritional intake), the extent to which energy and a wide range of nutrients in ONS add or displace those in the diet, and the extent to which they increase total intakes, allowing dietary reference values to be met.

## 2. Materials and Methods

### 2.1. Overview

The study was based on a prospective, randomised, parallel, open-label trial. Details of the full study and methodology, including the randomisation procedure, primary and secondary outcomes, full details of measurements and timeframes, and sample size calculations are described elsewhere [18].

### 2.2. Participants Selection and Ethics

In brief, 308 free-living older people were recruited from 179 general practice surgeries across 8 counties in England (UK), which took place between December 2012 and January 2017. Individuals were eligible to participate if they were aged >50 years, had disease-related malnutrition (according to the Malnutrition Universal Screening Tool (‘MUST’)), were able to eat, drink and provide informed consent. Nutritional supplementation was an exclusion criterion (see full trial paper [18]). Ethical approval was obtained from Southampton Central Research Ethics Committee A and the trial was conducted in accordance with their ethical standards and the Declaration of Helsinki. All subjects gave their informed consent for inclusion before they participated in the study. This trial was registered with the ISRCTN database on 3 October 2012. Registration number ISRCTN26004104.

### 2.3. Types of Intervention

**Figure 1:**
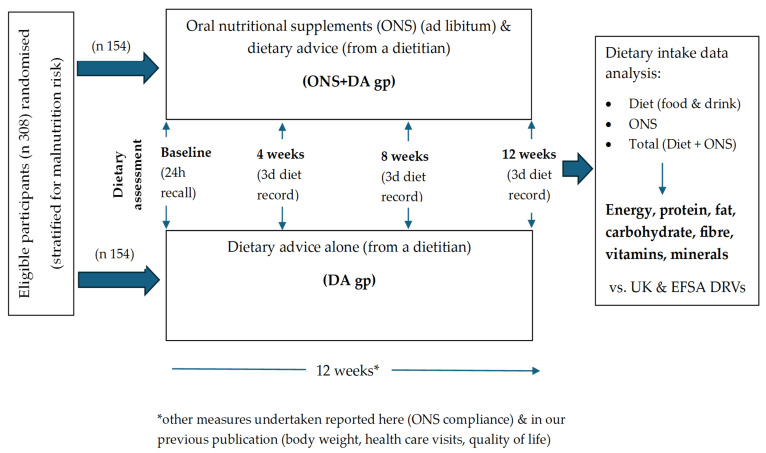
Scheme of intervention groups and dietary assessments. DRV: Dietary Reference Values; EFSA: European Food Safety Agency; ONS: Oral nutritional supplements; DA: Dietary advice.

Participants (stratified for malnutrition risk; medium or high risk) were randomised to receive either dietary advice alone (‘DA’ group) or the same dietary advice plus ready-to-drink ONS (‘ONS + DA’ group) (Figure 1). Dietary advice about food and drinks was given individually to each participant by a dietitian, and involved a widely used diet sheet, ‘Eating Well with a Small Appetite’ (NAGE, Older People Specialist Group, British Dietetic Association). Those in the ONS + DA group received additional guidance to consume high energy and/or high protein, low volume readymade ONS ad libitum (2.4 kcal/mL; Fortisip Compact or Compact Protein, Nutricia Ltd., Trowbridge, Wiltshire, UK). An alternative juice-type ONS (1.5 kcal/mL; Fortijuce, Nutricia Ltd.) was offered to those with an intolerance/dislike of milk. Participants were given a daily target of at least 600 kcal and 16 g protein (250–400 mL) for 12 weeks, although intake was voluntary, and participants remained in the trial irrespective of the quantity of the ONS consumed.

### 2.4. Data Collection and Outcomes

All data were collected during home visits at baseline (before intervention) and at weeks 4, 8 and 12 (±2 days). Medical history, age, gender, Charleston comorbidity index (CCI) and height (to the nearest 1 cm using a Leicester Height Measure) were recorded at baseline. Body weight (to the nearest 0.1 kg using calibrated Marsden ‘MS-4202 L’ scales), body mass index (weight/height^2^), percentage unintentional weight loss and ‘MUST’ score were measured and/or calculated.

### 2.5. Dietary Intake Assessment

At baseline, a dietitian assessed dietary intake by 24 h diet recall, involving the intake of all foods and drinks. At weeks 4, 8 and 12, participants were instructed to complete a 3-day diet record for the 3 days prior to the dietitian’s visit (Figure 1). All foods and drinks were recorded using household measures, and a checklist was used to ensure that all appropriate items were included. The participants were encouraged to provide as much detail as possible, including brand names of foods, adding any labels if they had them. The diet records were reviewed by the dietitian together with the participant, and any uncertainties or queries were discussed. If a participant had not completed the 3-day diet record, the dietitian obtained 24 h dietary recalls from them.

### 2.6. Compliance

Compliance to ONS, which was assessed by the dietitian, was calculated as the amount of ONS consumed expressed as a proportion of that advised (600 kcal). Compliance to DA was assessed simply by asking participants to provide a yes/no answer about whether they had made any dietary changes.

### 2.7. Dietary Intake Analysis

The dietary intake data were analysed using WISP (version 4, Tinuviel, Anglesey, UK). WISP’s food composition databank is based on McCance and Widdowson’s, ‘The Composition of Foods’ [19] (plus published supplements, the earlier 5th Edition and updates from the 7th edition). The intake of energy and a wide range of nutrients (protein, fat, carbohydrate, vitamins and minerals) during the intervention period was calculated using average daily intakes from diet alone, from ONS alone or from diet + ONS (total intake).

Total intakes were benchmarked against UK [20] and European (European Food Safety Authority (EFSA)) [21] dietary reference values (DRVs). Each DRV was specified either as population reference nutrient intakes (population reference intake) (RNI or PRI; the intake that is adequate for virtually all people in a population group) or adequate (safe) intake (AI; when the reference nutrient intake could not be established). The proportion of people not meeting these DRVs was assessed for comparison between ONS + DA and DA groups. Average requirements (for meeting the requirements of half the population) and reference intake ranges for macronutrients [21] were not analysed.

### 2.8. Dietary Intake Analysis: Equations and Modelling

(i) Equations to assess the relationship between ONS intake and percent of ONS nutrients that add to the diet

The equations used for calculating the net increase in nutrient intake with ONS + DA and DA alone and the percent of ONS intake that adds to dietary intake are as follows:

The equations (eq) assume there were two matched groups, one receiving ONS + DA, represented by superscript A (^A^) and the other receiving DA only (no ONS at all), represented by superscript B (^B^):

Net increase in nutrient intake due to ONS = Diet ^A^ + ONS intake ^A^−Diet intake ^B^ (1)

It follows:

% ONS nutrient that adds to Diet intake = 100 × (Net increase in nutrient intake/ONS intake ^A^) (2)

% ONS nutrient that displaces Diet intake = 100 − the result from Equation (2)(3)

For example, if the ONS + DA gp consumed 1820 kcal from the diet and 480 kcal from the ONS, their total intake would be 2300 kcal. If the matched DA group consumed 1848 kcal in total from the diet, the ONS + DA group would have consumed 28 kcal less from the diet (1848–1820 kcal). Therefore, the net increase in nutrient intake due to ONS is 452 kcal (480–28 kcal; i.e., the kcal of the ONS that are additive to the diet). This can then be expressed as a percentage of ONS that adds to the diet (100 × (452/480) = 94%).

Net increase in nutrient intake due to ONS = (1820 kcal + 480 kcal) − 1848 kcal (Equation (1)) = 452 kcal

% ONS nutrient that adds to diet intake = 100 × (452 kcal/480 kcal) (Equation (2)) = 94%

Although different units are used to express the energy and nutrient content of ONS (e.g., kcal, kJ, g, mg, μg, and ‘units’ or ‘equivalents’ in the case of certain vitamins), they can all be compared to each other by expressing ONS intake as a percentage of dietary intake (dietary equivalence). Equation (2) remains valid if each of the two terms on the right side of the equation is divided by diet intake^A^ (Equation (4)) or diet intake^B^ or the average of the two.

(4) $$\%\;\mathrm{ONS}\;\mathrm{nutrient}\;\mathrm{that}\;\mathrm{adds}\;\mathrm{to}\;\mathrm{diet}\;\mathrm{intake}=\frac{Net\;increase\;in\;nutrient\;intake}{Diet\;{intake}^{A}}/\frac{ONS\;{intake}^{A}}{Diet\;{intake}^{A}}$$

(ii) Dose-response modelling to assess the relationship between ONS intake and percent of ONS nutrients that add to the diet:

A type of dose-response curve involving energy and a wide variety of nutrients was constructed. The dose of ONS ingested (with each nutrient expressed as a dietary equivalent) was related to the % of ONS energy and nutrients that add to dietary intake (the response).

The above equations were used to construct such a dose-response curve using data from this study and compared with a series of theoretical dose-response curves (also based on the above equations) in which ONS-induced suppression of dietary intake was varied by fixed amounts up to 5%.

The above equations assume that:

- the two groups (ONS + DA and DA) had the same nutrient intake at baseline. (In the present study, the post-intervention results were adjusted for baseline nutrient intake);
- ingestion of ONS by the ONS + DA group (group A) during the intervention did not affect the composition of the diet, even when intake is suppressed (or stimulated)
- there was no ONS intake in the DA group. However, if the observed data from the randomized trial involved prescription of a small amount of ONS in the DA group for clinical reasons, the extra ONS (ONSe) consumed by the ONS + DA group (ONSe = ONS^A^ − ONS^B^) was used in the above equations (instead of ONS intake^A^), as an approximation to the model.

Lastly, although the above equations were applied to the measured intake of energy and most nutrients from the randomized trial, they were not applied to the following dietary constituents: fibre and fluoride (often regarded as non-essential dietary constituents); vitamin D, which has variable and usually unknown cutaneous synthesis following exposure to daylight; and chromium and molybdenum.

### 2.9. Statistics and Data Analysis

Details of the sample size calculations and full statistical methods are presented elsewhere [18].

Dietary intake data without adjustment for covariates or confounding variables were analysed using Chi-squared tests or Fisher exact tests (categorical variables), and *t*-tests (continuous variables). Analyses involving adjustment for covariates or confounding variables involved the general linear model (univariate analysis of variance, full factorial), which was used to adjust for baseline dietary intake values, ‘MUST’ category, age, gender, and CCI. Intention-to-treat analysis (ITT) was undertaken following multiple imputation that generated 5 sets of imputed data. Pooling of data was undertaken using Ruben’s rules [22], and the associated degrees of freedom (https://stats.oarc.ucla.edu/wp-content/uploads/2016/02/multipleimputation.pdf, accessed 5 August 2024) and *p*-values calculated using the most conservative option for the degrees of freedom.

Results are presented as mean ± SE, unless otherwise stated. The detailed results in Tables are based on ITT analyses, but comparisons were made with per protocol (PP) analyses. These analyses and imputations were undertaken using SPSS version 22.0 (Chicago, IL, USA). Curve fitting was undertaken using GraphPad Prism 10 (Boston, MA, USA).

## 3. Results

### 3.1. Population Characteristics

308 free living people >50 years were recruited (71.5, sd 10.7 years; 67% female) with a medium (44%) and high (56%) malnutrition risk (MUST). A total of 154 were randomised to receive ONS plus DA and 154 to receive DA only. At baseline, no significant differences were seen between the ONS + DA and DA groups respectively for gender (66%; 68% female), age (71.3; 71.6 y), weight (51.9; 52.3 kg), height (1.64 m; 1.64 m), BMI (19.3; 19.5 kg/m^2^), CCI (0.94; 1.09) and number of health problems per subject (2.62; 2.45). The main health problems were respiratory (34%), gastrointestinal (29%) and musculoskeletal (13%).

Over the 12-week intervention period, there were 69 (22.4%) dropouts. There were no significant differences in baseline characteristics between those that dropped out and those who did not, or between the dropouts from the ONS + DA and DA groups (refer to our previous publication for full details of recruitment and follow up dates, and participant flow (numbers randomized, receiving intended treatment, analysed, losses, exclusions etc.), adverse events [18]).

The majority of the ONS + DA group received a readymade high-energy, high protein, low volume ONS (2.4 kcal/mL) without fibre, although a small proportion (6%) did not like/tolerate milk and therefore received a juice-type ONS (1.5 kcal/mL). Of note, 4 participants in the DA group also received ONS, for clinical reasons. Despite this, the group remained the DA group to represent its classification at the time of randomisation.

### 3.2. Nutritional Intake at Baseline (ITT Analysis)

Table 1 shows that the baseline intake of energy and all the listed nutrients did not differ significantly between the ONS + DA and DA groups. The biggest discrepancy occurred with vitamin A, but due to wide variability within groups, the *p*-value was not significant (0.095). The normalised or standardised error for vitamin A (dividing the standard deviation, or standard error when N did not vary, by the mean) was found to be 4.85 (sd 1.44) times greater than the average of all the other 28 nutrients, and 7.0 times greater than that for energy.

### 3.3. Nutritional Intake (Average) from the Diet During the Intervention Period (ITT Analysis)

In response to dietary advice, changes to the diet were made by 66% of people in the DA group and 43% in the ONS + DA group. As in the baseline period, the dietary intake from food (not ONS) during the 12-week intervention period did not differ significantly between groups (even if further adjustments were made for dietary energy intake) (Table 2). Furthermore, the standardised errors for vitamin A intake remained large: 3.28 (sd 0.86) times greater than the average of all the nutrients for both groups combined.

### 3.4. Nutritional Intake (Average) from ONS During the Intervention Period (ITT Analysis)

Compliance to ONS was 80%. Table 2 shows that the intake of energy and nutrients from ONS was many times greater in the ONS + DA group than in the DA group (where a few participants received ONS for clinical reasons). For energy, it was 9.35 times greater (480 vs. 51 kcal/day), for protein 8.2 times greater and for all the listed nutrients except fibre, 6.89 (sd 0.91) times greater.

#### 3.4.1. Contribution of the Nutrients from ONS to Total Dietary Intake

**Figure 2:**
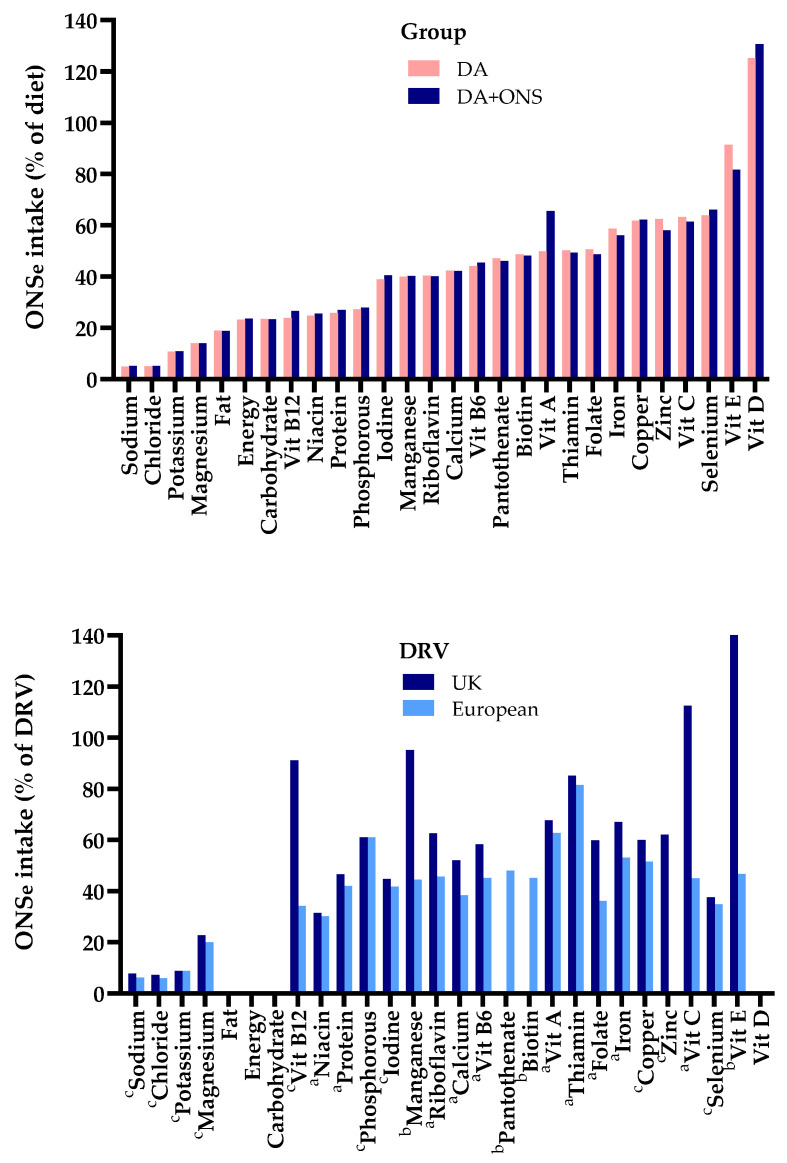
(**Upper**) Intake of nutrients from ONS (see Table 2) expressed as % of those in the diet of the DA and ONS + DA groups. (**Lower**) Percent of UK and European DRVs met by intake of ONS alone (excluding fibre and potential pantothenate). ^a^ Dietary reference values (DRVs) classified as reference nutrient intakes (RNI) by the UK and Europe; ^b^ DRVs classified as adequate (safe) intakes (AI) by the UK and Europe, or Europe alone; ^c^ DRVs classified as RNIs by the UK and AIs by Europe. Vit: Vitamin. ONSe = ONS intake of the ONS + DA gp calculated as over and above the small amount ingested by the DA group.

The contribution of ONS nutrients ingested as a proportion of the total dietary intake varied widely for the ONS + DA group, depending on the nutrient (see Figure 2 upper; calculated as over and above the small amount ingested by the DA group). For sodium and chloride, the intake from ONS was equivalent to only 5% of that in the diet (5% dietary equivalence), for several other nutrients it was more than 50% (iron copper, zinc, selenium and vitamins C, E), and for vitamin D it was over 100% (Figure 2 upper).

The average intake from ONS of all 28 individual nutrients (excluding fibre) was equivalent to 43% (sd 26%) of the dietary intake of the ONS + DA group. For ONS energy intake and the intake of macronutrients (protein, fat and carbohydrate), the contribution (equivalence) was smaller, ranging from 19% to 24% of the dietary intake of the ONS + DA group. For trace elements and vitamins (*n* = 19), the contributions from ONS were larger, averaging 54% of dietary intake of the ONS + DA group.

#### 3.4.2. Contribution of the Nutrients from ONS to Meeting Dietary Reference Values

In Figure 2, lower shows that the nutrients from the ONS frequently accounted for a large proportion of the amounts required to meet the DRVs. On average ONS provided 40% (sd 18%) of the individual European DRVs (23 nutrients) and 59% (sd 36) of the UK DRVs (22 nutrients).

The vitamins in ONS provided an even greater proportion of the European (47% (sd 12%; *n* = 16) and UK DRVs (73% (sd 33%; *n* = 15), while protein accounted for a much smaller proportion (22% of the European RNI and 31% of the UK RNI). (The legend of Figure 2 distinguishes between DRVs that are population reference intakes (RNI) and the adequate (safe) intakes (AI)).

### 3.5. Total Nutritional Intake (ONS and Diet) (Average) During the Intervention Period (ITT Analysis)

#### 3.5.1. Comparison of the Total Nutrient Intakes Between the ONS + DA and DA Groups

Table 2 shows that total intake in the ONS + DA group was significantly greater than in the DA group for energy and most (25 out of 29) nutrients (by a mean of 42% (sd 22%), except for fibre, sodium, chloride, and vitamin B_12_ (which had a *p*-value of borderline significance (*p* < 0.06)).

For energy, the mean intake was 21% higher in the ONS + DA group than the DA group, and for 29 individual nutrients it was 37% (sd 24%) higher but with large variation between nutrients (−4% (fibre) to over 100% higher (vitamin D); 1% to over 100% higher without fibre).

The intake of the three macronutrients, protein, fat and carbohydrate in the ONS + DA group exceeded that of the DA group by a mean of 21% (sd 2%). For the other 26 nutrients, the ONS + DA group intakes exceeded that of the DA group by a mean of 40% (sd 24%) (and by a mean of 47% (sd 21%) for the subgroup of 19 trace elements and vitamins).

**Figure 3:**
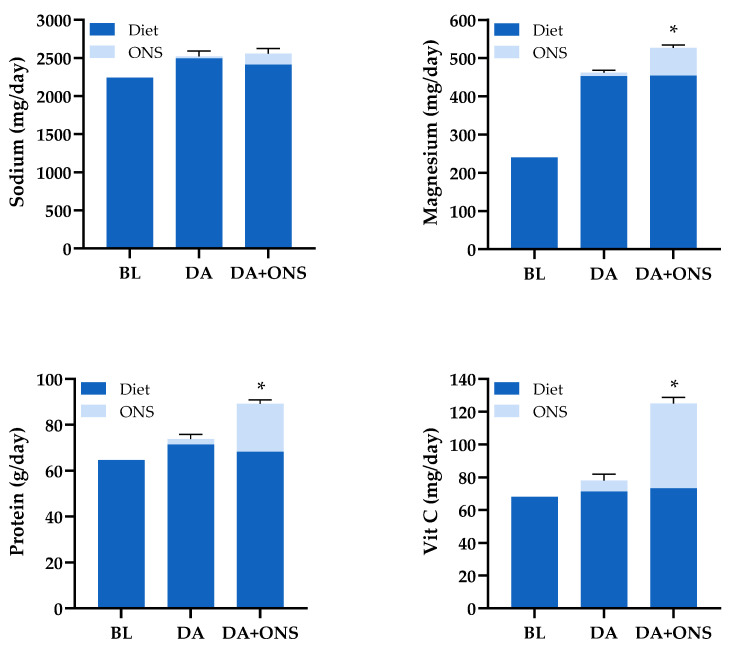
Average total daily intake of selected nutrients during the intervention period by group, adjusted for baseline intake (BL) (see Table 2). The contribution of ONS and diet to total intake, shown as the stacked bars, varies substantially with the nutrient: (**top left**) sodium, with the smallest ONS contribution to total intake; (**top right**) magnesium (typical of non-trace element minerals); (**bottom left**) protein (typical of energy and energy containing macronutrients); (**bottom right**) Vitamin C (typical of trace-elements and other vitamins). The asterisks at the top of the standard error bars for total intake indicate significantly greater intake (*p* < 0.001) in the ONS + DA group than the DA group.

Figure 3 shows that the extent to which the total intake of selected nutrients is increased by supplementation with ONS is far greater for some nutrients than others.

#### 3.5.2. Comparison of Total Nutritional Intakes with Dietary Reference Values

**Figure 4:**
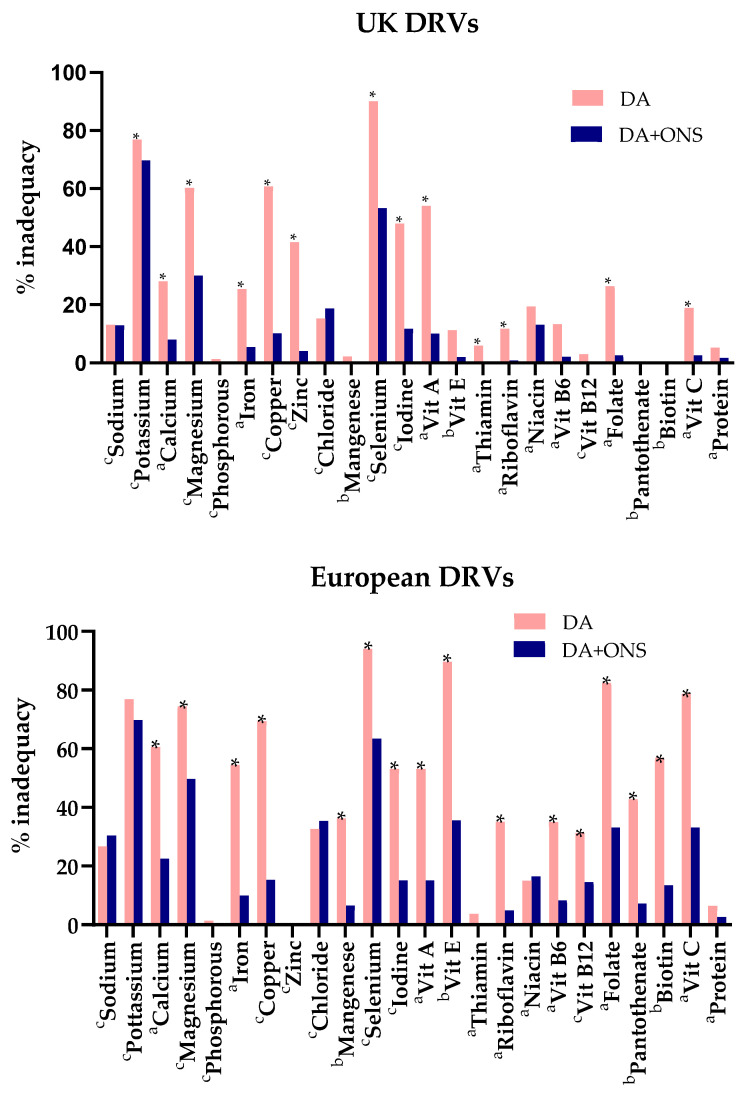
Percentage of subjects in the ONS + DA and DA groups not meeting the UK and European DRVs. Significant differences between groups (Fisher’s exact test) are indicated (*p* < 0.05 *). ^a^ Dietary reference values (DRVs) classified as reference nutrient intakes (RNI) by the UK and Europe; ^b^ DRVs classified as adequate (safe) intakes (AI) by the UK and Europe, or Europe alone; ^c^ DRVs classified as RNIs by the UK and AIs by Europe. DA: dietary advice; ONS: oral nutritional supplements; Vit: vitamin.

Failure to meet the UK and European DRVs (RNI and AI) was significantly greater in the DA group than the ONS + DA group for most nutrients (Figure 4). On average, 26% more people in the DA group than the ONS + DA group did not meet the individual European DRVs (48% vs. 22%, 22 nutrients; compared to 29% vs. 12% for the UK DRVs, 22 nutrients).

For vitamins and trace elements, the results were amplified with an average of 35% more subjects in the DA group than the ONS + DA group failing to meet individual European DRVs (52% vs. 17%; 16 nutrients; compared to 29% vs. 8% for the UK DRVs; 16 nutrients).

None of the subjects met the European adequate safe intake for vitamin D (15 μg/day) at baseline or during the intervention period in either group, although this excludes the unknown quantity synthesized in the skin (only one subject in the ONS + DA exceeded 10 μg/day during the intervention period, corresponding to the UK RNI for those >65 y).

For protein (g/kg body weight), the differences were smaller and not significant, both when benchmarked against the European RNI (6.4% vs. 2.6%) and UK RNI (5.2% vs. 1.7%).

The number of nutrients per person failing to meet the DRVs was more than halved by supplementation (DA vs. ONS + DA group for European DRVs, 11.7 vs. 5.0; and 7.7 vs. 3.4, for UK DRVs). Within the supplemented group (ONS + DA) an increase in the intake of individual nutrients from ONS correlated with the number of people meeting individual DRVs, with r values ranging from 0.208 to 0.534 (mean r, after back transforming the mean Fisher r′, was calculated to be 0.410 (+1sd, 0.082, and −1sd, 0.120); *n* = 154 subjects for each of the 21 nutrient correlations). Increasing ONS nutrient intake from ONS in the ONS + DA group also correlated with the number of nutrients per person meeting the DRVs.

#### 3.5.3. Net Increase in Intake with Supplementation (% ONS That Adds to Dietary Intake)

**Figure 5:**
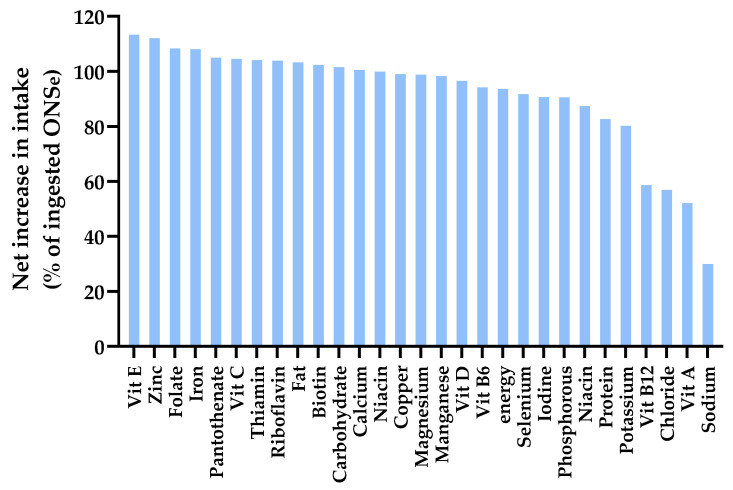
Bar chart showing the net increase in energy and nutrient intake expressed as % of ingested ONS Net increase in intake = total intake by ONS + DA group minus total intake by DA group. ONS: oral nutritional supplements; DA: dietary advice. Calculated using ONSe = the extra ONS energy or nutrients ingested by the ONS + DA group compared to the ONS intake in the DA group.

The extent to which the nutrients in ONS added to dietary intake varied (Figure 5). The net increase averaged 92% of the amount in the ONS, with the equivalent of only 8% displaced from the diet. For energy, the value was 94% (only 6% dietary displacement). For 22 out of the 29 nutrients, more than 90% of the amounts present in ONS are added to dietary intake (<10% displaced from the diet). The lowest values were for sodium, chloride, vitamin A and vitamin B_12_ (Figure 5).

#### 3.5.4. Dose-Response Modelling to Assess the Relationship Between ONS Intake and Percent of ONS Nutrients That Add to the Diet

**Figure 6:**
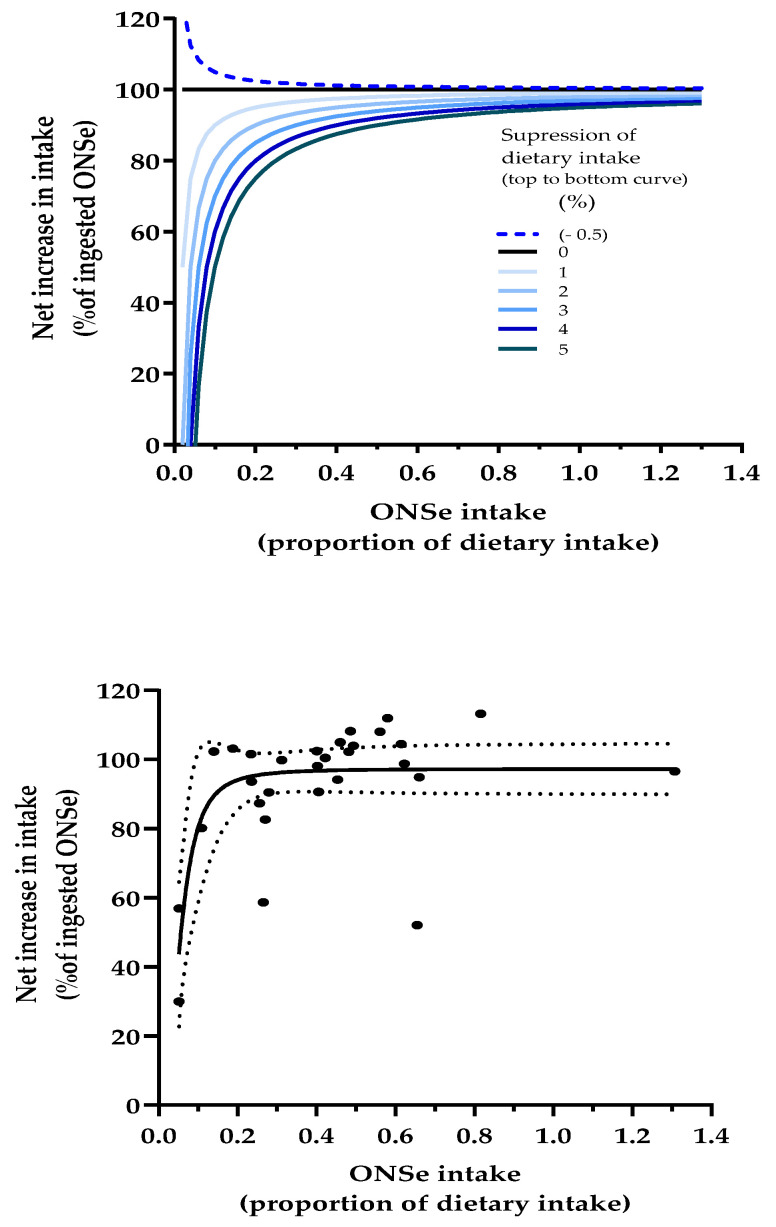
(**Upper**) Theoretical dose-response curves showing how the net increase in intake varies with ONS intake and suppression of dietary intake (ONS + DA group) by 0–5% (or stimulation by 0.5%). (**Lower**) Observed dose-response curve for energy and 28 nutrients (fibre is excluded). The dotted lines represent the asymmetric 95% confidence interval. The outlier in the middle of the graph is vitamin A. The data in both the (**upper**) and (**lower**) graphs were analysed using the same inbuilt dose-response graphical software model (GraphPad agonist-response, variable slope model, employing no constraints apart from zero bottom), yielding a near perfect relationship between dose and response for the theoretical upper curves (R = 0.993–1.000) and a less strong relationship for the observed lower curve (R = 0.703). Calculated using ONSe = the extra ONS energy or nutrients ingested by the ONS + DA group compared to the ONS intake in the DA group.

Figure 6 (upper) shows theoretical dose-response curves that define the extent to which the ONS intake consumed (dose) adds to or displaces energy and nutrients from the diet (expressed as % of ONS intake) (response). The dose-response curves range from −0.5% (i.e., 0.5% stimulation of dietary intake) through to 5% (displacement of dietary intake), as the intake (dose) of ONS is increased.

Figure 6 lower shows the observed dose-response curve for energy and 28 nutrients (fibre is excluded) and includes the low values for ONS sodium and chloride (representing only ×0.05 (5%) of dietary intake) and the high value for ONS vitamin D intake (representing up to ×1.3 (130%) of dietary intake).

All the curves rise steeply as ONS intake increases between 0 and ×0.2 as a proportion of dietary intake, before beginning to plateau by converging towards 100%. Higher ONS intakes and smaller suppression of dietary intake elevate the points on the curves. Figure 6 (lower) shows that the observed dose-response curve for energy and nutrients follows the same general trajectory as the curves on the upper graph. However, the curve was strongly influenced by sodium and chloride intake (the two most left points on the graph, with a dietary equivalence of only 5%), and vitamin A intake, the most extreme outlier, shown in the middle of the graph.

### 3.6. Nutritional Intake (Average) from the Diet: Per Protocol (PP) and Intention to Treat (ITT) Analyses Compared

As with the ITT analysis, the PP analysis during the intervention period showed no significant differences between groups in the dietary intake of energy or any of the nutrients (except for selenium, which favoured the DA group in the PP analysis). There was also large variability in vitamin A intake during the intervention period, with standardised errors 4.05 (sd 1.07) times greater than the average of all other 28 nutrients for both groups combined.

### 3.7. Nutritional Intake (Average) from ONS: Per Protocol (PP) and Intention to Treat (ITT) Analyses Compared

Compliance to ONS (ONS + DA group) was found to be 83% by PP analysis and 80% by ITT analysis.

Both the PP and ITT analyses showed similar results. As with the ITT analyses, the PP analyses showed that the dietary equivalence of ONS was about two times greater for trace elements and vitamins than for the energy-containing macronutrients. These in turn were about 1.3 times greater than those for the remaining nutrients (the non-trace element minerals: sodium, potassium, chloride, calcium, magnesium, phosphorus).

### 3.8. Total Nutritional Intake (ONS and Diet) (Average): Per Protocol (PP) and Intention to Treat (ITT) Analyses Compared

In both PP and ITT analyses, the total intake of energy and nutrients were all significantly greater in the ONS + DA group than the DA group, except for sodium, chloride and fibre (potassium and vitamin B_12_ were nonsignificant only in the ITT analysis, although vitamin B_12_ was of borderline significance in the ITT analysis (*p* < 0.06)).

#### 3.8.1. Comparison with Dietary Reference Values

Overall, the average proportion of people not meeting the individual European DRVs (23 nutrients) was 50% by the PP analysis and 48% by ITT analysis, compared to 28% (PP analysis) vs. 29% (ITT analysis) of people not meeting the UK DRVs.

Both PP and ITT analyses showed that the proportion of people not meeting the DRVs varied considerably according to the nutrient, but with similar results for the individual nutrients by both types of analysis.

The average proportions not meeting the European DRVs for 22 individual nutrients were (PP vs. ITT) 51% vs. 48% for the DA group, and 21% vs. 22% for the ONS + DA group. The corresponding values for the UK DRVs (22 nutrients) were 31% vs. 29% for the DA group and 11% vs. 12% for the ONS + DA group.

The paired differences for individual nutrients were small, averaging (PP-ITT) as follows: for European DRV benchmarking, −0.9 ± 1.0% (sd) for ONS + DA group, and 2.6 ± 3.3% for the DA group; and for UK DRV benchmarking, −0.4 ± 0.8% for the ONS + DA group and 1.9 ± 3.0% for the DA group. There was no significant relationship between the difference and the average of the two groups.

The average number of nutrients per subject not meeting the DRVs was greater in the DA group than the ONS + DA group, both by PP and ITT analyses (PP vs. ITT): for the European DRV benchmarking, 11.2 vs. 11.7 for the DA group, and 4.8 vs. 5.0 for the ONS + DA group; and for the UK DRV benchmarking: 7.7 vs. 7.4 for the DA group and 3.4 vs. 3.5 for the ONS + DA group.

**Figure 7:**
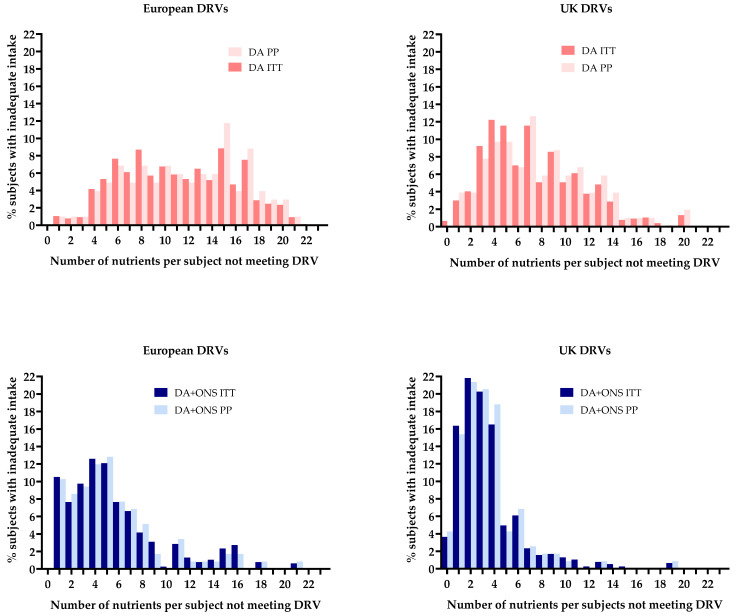
The frequency (% of subjects with inadequate intake) of the number of nutrients not meeting DRV per person by group (DA and DA + ONS) and DRV origin (UK and European) by ITT and PP analysis. DRV: dietary reference values; ITT: intention to treat; PP: per protocol.

The histograms in Figure 7 reflect these results and show generally consistent outcomes between PP and ITT analyses. There were also similarities in distribution, including a shift to the left in the ONS + DA group (lower graphs), representing fewer nutrient intake inadequacies per person in this group compared to the DA group (upper graphs).

#### 3.8.2. Dose-Response Modelling to Assess the Relationship Between ONS Intake and Percent of ONS Nutrients That Add to the Diet

A comparison of the ITT and PP analyses that generated the dose-response curves revealed greater discrepancies than the other analyses described above. The overall mean difference between PP and ITT for the % of ONS intake that added to dietary intake was 5.6% with a standard sd of 14% (28 nutrients). The most striking differences concerned sodium (PP vs. ITT; 78% vs. 30%) and chloride (83% vs. 57%), which had influential effects on the shape and characteristics of the dose-response curve. Compared to the ITT dose-response curve shown in Figure 6, the PP curve lost its steep initial rise and began as a flatter curve above a value of about 80%, with some poorly defined 95% confidence interval profile characteristics, and an overall R value of 0.245. Vitamin A remained an outlier in the PP analysis, although by 11% less than in the ITT analysis.

## 4. Discussion

This community-based study of malnourished older people found that readymade low-volume liquid ONS significantly increased the total intake of most nutrients, with typically little or no displacement of nutrients from the diet. Since the use of ONS in the ONS + DA group more than halved both the number of people failing to meet individual DRVs and the number of nutrients per person failing to meet DRVs, this form of nutritional support intervention can be regarded as improving the quality of total nutritional intake. Furthermore, since the amount of ONS ingested within the supplemented group correlated with the number of subjects meeting the DRVs, the finding provides a rationale for encouraging ONS intake in those with poor compliance with the ONS prescription.

### 4.1. Impact of ONS on Total Nutrient Intake and Meeting DRVs

Although the nutritional effects of ONS are often considered in terms of energy and protein, appropriate nutritional support aims to provide adequate and balanced amounts of many nutrients. In this study, the consumption of energy and energy-containing macronutrients (protein, carbohydrate and fat) in ONS was equivalent to 19–24% of dietary intake. This was generally about half of that of the trace elements and vitamins from ONS, which made a far greater contribution to dietary intake (equivalent to approximately 54% of dietary intake). Furthermore, only about 6% of the non-supplemented group failed to meet the European RNI (PRI) for protein (5% for the UK RNI). Supplementation with an extra 15 g protein/day (21% increase), although reducing this percentage in the ONS + DA group 2–3 times, did not reach significance versus the DA group (ITT analysis). In contrast to protein, on average, almost 8 times more subjects in the DA group failed to meet the European DRVs for individual trace elements and vitamins (>5 times more for the UK DRVs), while ONS use typically significantly reduced this frequency to less than half.

When all 22 nutrients were individually benchmarked against the European DRVs (RNIs and AIs), on average, 26% more of the total number of people in the DA group than the ONS + DA group did not meet the DRVs (48% vs. 22%). For trace elements and vitamins, it was 34% more subjects in the DA than ONS + DA group, and for specific vitamins, such as vitamins C and E, and specific trace elements, such as iron and copper, the impact of ONS was even more striking. Benchmarking against the UK DRVs also revealed that supplementation with ONS attenuated inadequacies in nutrient intake, but since the UK DRVs are lower than the European DRVs for most nutrients, the frequency of the inadequacies was lower.

### 4.2. Benchmarking the Total Intake of Our Study Population Against the UK and European

Benchmarking with DRVs should be interpreted with some caution because both DRVs were developed for healthy populations rather than specifically for those with disease-related malnutrition. However, at the very least, the study shows that ONS supplementation produces a net increase in intake that is equivalent to a substantial proportion of many nutrient DRVs. Caution should also be exercised in extrapolating the specific nutrient results of this ONS supplementation study to other supplementation studies that use different commercial ONS products that vary in their nutritional composition, palatability and usability. In this study, the only nutrient from ONS that exceeded that from the diet was vitamin D. Although the total intake was less than half of the European PRI, additional vitamin D can be provided by cutaneous synthesis.

### 4.3. Energy/Nutrient Displacement from the Diet and % ONS That Adds to Dietary Intake

This study in malnourished free-living older people found that dietary energy compensation was equivalent to only 6% of ONS energy, leaving 94 % additive to dietary intake. This contrasts with much greater compensation reported in non-malnourished subjects participating in preload studies, where food [23] or ONS [24,25] is ingested before meals. There are at least four possible explanations for these discrepancies. First, liquid supplements could be less satiating than solid supplements. A systematic review of preload studies in populations ranging from lean to overweight/obese subjects concluded that liquid preloads are least compensated for energy, followed by semi-solid and solid preloads [23]. Second, chewing, which is reported to reduce appetite and dietary intake [26], is not used when drinking liquid supplements. Third, fibre, which may reduce satiety [27] (at least partly by increasing viscosity and slowing gastric emptying [28]), was almost totally absent from the ONS used in this study.

The fourth possibility, tentatively implied from our previous work [6], is that disease-related malnutrition modifies the response observed in health. Compared to the 6% ONS energy compensation observed in this study of subjects with disease-related malnutrition, a review of preload studies involving non-malnourished subjects found that the average (median) compensation was 43% (57% additive to diet) for liquid preloads, and 62% (38% additive) for composite meal preloads. Furthermore, when a liquid ONS, like the one used in the present study, was administered as a preload for 5 days to a small group of lean but healthy women, there was 43% ONS energy compensation (57 % added to dietary intake) [24], and in a similar earlier study [25] even greater ONS energy compensation was reported (54%) [24]. In addition, a small study directly comparing responses between malnourished and healthy subjects found that the same preload, in the form of ice cream, produced no suppression in dietary intake in elderly malnourished subjects, but substantial suppression in both older and younger healthy subjects [29]. When the reported results were recalculated as ice cream equivalents (by the authors of the present manuscript), it was found that a little over 100% of ice cream energy was added to the dietary intake of older malnourished subjects, compared to 50% in healthy older subjects and 44% in younger healthy subjects. Furthermore, the specific use of liquid ONS as a preload in a small experimental study involving patients (some with malnutrition) recovering from hip fracture found that ONS ingestion 30 and 90 min before meals resulted in an average energy compensation of only 6.3% (93.7% added to dietary intake) [30].

Our longer-term community study presented in this manuscript had the same result for energy as the above study [30], and a comparable small overall displacement of nutrients from the diet. This is encouraging with respect to the clinical use of readymade liquid ONS in the community. Supplementation overcomes many nutrient intake inadequacies without significantly reducing intake from the usual diet. This conclusion is consistent with another study of 30 malnourished patients with cardiopulmonary disease, who were studied before and after discharge from hospital [17]. However, in that study, the extent to which individual or groups of nutrients in ONS added to dietary intake was not evaluated [17]. Similarly, other studies have also reported increases in total nutrient intake with ONS, but have not calculated the degree to which ONS nutrients displace or add to the diet [12,13,16,31,32,33,34]. Furthermore, it is not possible to undertake the relevant calculations from such studies without distinct information on ONS and dietary intake, which is typically missing from such studies.

The present study has extended the concept of energy compensation and dietary energy displacement with ONS to a wide variety of nutrients. This study found dietary equivalents for ONS intakes ranging from 5% (sodium and chloride) to over 100% (vitamin D). The observed ITT ONS dose-response curve showed the extent to which increasing the dose of different ONS nutrients (expressed as dietary equivalents) produced an increasing response in net total intake (expressed as % of ONSe intake). This was found to be generally consistent with the theoretical dose-response curves, although it was disproportionately influenced by certain data points. One of these was vitamin A, which was not only an outlier on the dose-response curve, but also with respect to the difference in dietary intake between groups (Figure 2), and an anomalously high standardised error for dietary intake. The dietary intake of vitamin A is also unusual in that its within-subject variation is almost entirely due to day-to-day variation rather than methodological variation [35]. And, since the coefficient of variation for the dietary intake of vitamin A is far higher than other nutrients [36], it may take well over two months of dietary records to obtain a reproducible result within ±10% of the average intake. In this study, the average of three 3-day periods of dietary assessment by dietary recall is clearly inadequate to achieve such precision, which may explain why vitamin A is an outlier in several analyses. The other influential data points affecting the shape of the dose-response curve concern sodium and chloride, which have the lowest dietary equivalents compared to other nutrients (extreme points on the left of the curve; Figure 6 lower). Small measurement errors in dietary intake, even those that do not cause significant differences between groups, can become considerably amplified when expressed as a percentage of the very little sodium or chloride present in the ingested ONSe (the outcome measure of the dose-response curve), with resulting distortion of the shape and characteristics of the dose-response curve.

The model on which the dose-response curve was based assumes that there is no baseline imbalance in nutrient and energy intake. To avoid this potential problem, intake during the intervention period was adjusted for baseline intake, so that the same baseline dietary nutrient intake applied to both groups (Table 2). Another assumption of the model is that ONS did not alter the composition of the ingested diet. Although subjects in both groups reported making some dietary adjustments, no significant nutrient differences were found between groups, even after further adjustments for dietary energy intake (a procedure commonly used to adjust for ingestion of different amounts of diets). However, very small between-group differences in dietary intake of sodium or chloride, with potentially consequential effects on the sensitive part of the dose-response curve, cannot be excluded.

### 4.4. Limitations of Study

The randomised trial had an open-label design, which means that it was not blinded with respect to ONS administration. Dietary intake may have been better assessed using the weighed food intake methodology, but this was not considered practical for at least some of the study participants. Markers of nutrient status were also not measured. Like many other trials, dropouts occurred in this study, but it is reassuring that the overall study results and conclusions obtained by ITT analysis are strongly reinforced by PP analyses. The trial did not have a control arm that received no nutritional intervention (i.e., standard care) for comparison. However, it would have been difficult ethically to withhold nutrition in a group of older people identified with nutritional risk.

Although ONS, which are used under medical supervision, are a nutritional intervention that is typically safe to use, with very few risks or side effects [1,2,3,4,6], there are some patient groups for whom the ONS types used in this trial could be contraindicated or further complex dietary adaptations would be required beyond the scope of this trial. Therefore, these groups were excluded from recruitment (e.g., galactosaemia or known lactose intolerance, renal or liver failure, dysphagia and poorly controlled diabetes). Furthermore, those unable to consent (e.g., dementia) were also excluded. Consequently, further research in older people with such conditions may be warranted.

### 4.5. Clinical Applications for the Management of Malnutrition in Older People

This research [18], together with the findings of other randomised trials and systematic reviews [1,2,3,4,5,6], supports the valuable role of liquid ONS in the management of older people (aged over 50 y) at risk of malnutrition, helping to improve nutritional intakes and functional and clinical outcomes. ONS are indicated for those with disease-related malnutrition (unintentional loss of weight, muscle mass, thinness (low body mass index), functional decline) who cannot meet their nutritional requirements from the diet and require additional oral nutritional support (for whom oral intake is not contraindicated). This can be due to anorexia associated with disease, inflammation or the treatments of disease (surgery, pharmacotherapy) or other symptoms that limit food intake (e.g., dysphagia, breathlessness, gastrointestinal symptoms, etc.) [8]. To identify those that require ONS in clinical practice, routine screening for malnutrition should be undertaken on entry to health care settings using a validated tool. If required, a full nutritional assessment, including micronutrient status, body composition and functional measures, should be undertaken. As ONS are used in addition to diet, dietary advice to accompany supplementation is recommended, ideally with input from a dietitian, especially when there are complex conditions and nutritional needs. When prescribing nutritional support, clinicians should consider the modality that will most effectively improve the intake of all the nutrients (including vitamins, minerals and not just energy and protein), and not suppress appetite. Evidence from this trial, and others, suggests that liquid, more energy-dense formats have little suppressive effect on appetite and food intake, being largely additive (as opposed to displacing) to food intake in malnourished individuals [1,6,9,15]. Regular monitoring of compliance, intake, body weight and composition, and outcomes (relevant to the individual patient, e.g., muscle strength, quality of life, health care use) is recommended with all nutritional interventions to make sure they are used appropriately. For some patients, additional nutritional support may be indicated (such as enteral tube feeding or parenteral nutrition). Provision of suitable, easy-to-read and understand information on the nutritional interventions provided (e.g., dietary advice leaflets, ONS information (what they are, why they are of value, what to take, when and for how long) will help older people with malnutrition better understand their nutritional management.

## 5. Conclusions

In summary, this randomised trial in free-living malnourished older people shows that supplementation with readymade, low volume (2.4 kcal/mL) liquid ONS overcomes most nutrient intake inadequacies without significantly reducing intake from the diet. This enables older people to meet more of their individual reference nutrient intakes for a wide range of vitamins, minerals and trace elements than with food and dietary advice alone. These wide-ranging, significant improvements in nutritional intake with the use of ONS may be a key part of the mechanism by which ONS led to improvements in clinical outcomes observed in this and other trials, but further research is required.

## Figures and Tables

**Table 1:** Baseline (non-adjusted) daily intake of dietary constituents (mean ± SE; original data).

	ONS + DA	DA	ALL	*p*-Value ^a^
	*n* = 154 ^b^	*n* = 152 ^b^	*n* = 306 ^b^	
Energy (kcal)	1759 ± 46	1711 ± 46	1735 ± 32	0.465
Protein (g)	65.4 ± 1.7	64 ± 1.8	64.7 ± 1.2	0.579
Carbohydrate (g)	207 ± 6	206 ± 6	207 ± 4	0.918
Fat (g)	76.1 ± 2.7	71.1 ± 2.6	73.6 ± 1.9	0.182
Fibre (g)	15.2 ± 0.6	15.1 ± 0.6	15.1 ± 0.4	0.951
Sodium(mg/d)	2228 ± 78	2246 ± 106	2237 ± 65	0.893
Potassium (mg/d)	2726 ± 74	2654 ± 69	2690 ± 51	0.478
Calcium (mg/d)	820 ± 26	816 ± 31	818 ± 20	0.918
Magnesium (mg/d)	241 ± 7	239 ± 7	240 ± 5	0.852
Phosphorous (mg/d)	1152 ± 30	1144 ± 32	1148 ± 22	0.856
Iron (mg/d)	9.45 ± 0.32	10.02 ± 0.38	9.73 ± 0.25	0.254
Copper (mg/d)	1.05 ± 0.05	1.15 ± 0.14	1.1 ± 0.07	0.474
Zinc (mg/d)	7.56 ± 0.22	7.66 ± 0.27	7.61 ± 0.17	0.783
Chloride (mg/d)	3303 ± 113	3318 ± 151	3311 ± 94	0.934
Manganese (mg/d)	3.28 ± 0.12	3.27 ± 0.11	3.27 ± 0.08	0.957
Selenium (mcg/d)	37.2 ± 1.86	34 ± 1.6	35.6 ± 1.2	0.194
Iodine (mcg/d)	143 ± 8	137 ± 8	140 ± 6	0.599
Vitamin A (mcg/d)	489 ± 49	758 ± 154	623 ± 81	0.095
Vitamin D (mcg/d)	2.57 ± 0.28	2.29 ± 0.24	2.43 ± 0.18	0.448
Vitamin E (mg/d)	6.77 ± 0.36	6.25 ± 0.34	6.51 ± 0.25	0.292
Thiamin (mg/d)	1.37 ± 0.04	1.39 ± 0.06	1.38 ± 0.04	0.781
Riboflavin (mg/d)	1.67 ± 0.06	1.76 ± 0.06	1.72 ± 0.04	0.294
Niacin (mg/d)	15.9 ± 0.7	16.6 ± 0.8	16.3 ± 0.5	0.472
Potential niacin (mg/d)	13.4 ± 0.4	13.0 ± 0.4	13.2 ± 0.3	0.485
Vitamin B6 (mg/d)	1.63 ± 0.05	1.61 ± 0.06	1.62 ± 0.04	0.82
Vitamin B12 (mcg/d)	4.43 ± 0.25	4.86 ± 0.38	4.64 ± 0.23	0.337
Folate (mcg/d)	224 ± 7	235 ± 8	230 ± 5	0.283
Pantothenic acid (mg/d)	4.89 ± 0.16	4.98 ± 0.18	4.93 ± 0.12	0.696
Biotin (mcg/d)	35.9 ± 1.3	36.5 ± 1.3	36.2 ± 0.9	0.774
Vitamin C (mg/d)	72.5 ± 4.5	63.4 ± 4.2	68 ± 3.1	0.142

^a^ *p*-value (independent sample *t*-test: ONS + DA vs. DA group); ONS oral nutritional supplements; DA dietary advice. ^b^ Although 154 subjects were assigned to each group, there were two missing datasets in the DA group (*n* = 152), and 1 subject in the ONS + DA group had missing magnesium, manganese, niacin and biotin values (*n* = 153 for these nutrients).

**Table 2:** Comparison of the average energy and nutrient intake of the two groups during the 12-week intervention period (mean ± SE; ITT analysis) ^†^.

	Baseline	ONS + DA Group (*n* = 154)			DA Group (*n* = 154)		
	Diet	ONS Only	Diet Only	Diet + ONS	ONS Only	Diet Only	Diet + ONS
Energy (kcal/d)	1735	480 ± 12 ^b^	1820 ± 41	2300 ± 43 ^b^	51 ± 13	1848 ± 43	1899 ± 45
Protein (g/d)	64.7	21.0 ± 0.6 ^b^	68.2 ± 1.6	89.2 ± 1.7 ^b^	2.5 ± 0.7	71.4 ± 1.7	74.0 ± 1.9
Carbohydrate (g/d)	207	60 ± 2 ^b^	218 ± 5	278 ± 5 ^b^	9 ± 2	217 ± 5	226 ± 6
Fat (g/d)	73.7	16.9 ± 0.6 ^b^	76.3 ± 2.1	93.2 ± 2.1 ^b^	2.6 ± 0.6	75.8 ± 2.4	78.4 ± 2.4
Fibre (g/d)	15.18	0.02 ± 0.01	15.38 ± 0.43	15.39 ± 0.43	0.01 ± 0.01	15.94 ± 0.46	15.95 ± 0.47
Sodium (mg/d)	2243	149 ± 5 ^b^	2410 ± 63	2559 ± 64	25 ± 6	2497 ± 69	2521 ± 70
Potassium (mg/d)	2693	365 ± 14 ^b^	2831 ± 58	3196 ± 6 ^a^	58 ± 14	2892 ± 60	2950 ± 63
Calcium (mg/d)	819	417 ± 15 ^b^	862 ± 25	1279 ± 28 ^b^	54 ± 15	860 ± 27	914 ± 30
Magnesium (mg/d)	240	73 ± 2 ^b^	255 ± 6	328 ± 7 ^b^	9 ± 3	256 ± 6	265 ± 6
Phosphorous (mg/d)	1148	383 ± 14 ^b^	1203 ± 26	1586 ± 29 ^b^	48 ± 14	1234 ± 27	1282 ± 30
Iron (mg/d)	9.7	6.8 ± 0.2 ^b^	10.4 ± 0.3	17.2 ± 0.3 ^b^	1 ± 0.2	9.9 ± 0.3	10.9 ± 0.3
Copper (mg/d)	1.10	0.83 ± 0.02 ^b^	1.16 ± 0.06	1.99 ± 0.06 ^b^	0.11 ± 0.02	1.17 ± 0.06	1.28 ± 0.07
Zinc (mg/d)	7.61	5.6 ± 0.15 ^b^	8.36 ± 0.23	13.97 ± 0.27 ^b^	0.75 ± 0.16	7.78 ± 0.23	8.53 ± 0.27
Chloride (mg/d)	3314	216 ± 11 ^b^	3532 ± 86	3748 ± 88	36 ± 12	3610 ± 108	3645 ± 110
Manganese (mg/d)	3.28	1.54 ± 0.04 ^b^	3.32 ± 0.1	4.86 ± 0.1 ^b^	0.2 ± 0.04	3.34 ± 0.11	3.55 ± 0.12
Selenium (mcg/d)	35.8	28.3 ± 0.8 ^b^	37.0 ± 1.2	65.3 ± 1.4 ^b^	3.9 ± 0.8	39.1 ± 1.5	42.9 ± 1.6
Iodine (mcg/d)	140	73 ± 2 ^b^	155 ± 7	227 ± 7 ^b^	10 ± 2	160 ± 7	170 ± 8
Vitamin A (mcg/d)	629	498 ± 14 ^b^	654 ± 74	1152 ± 74 ^a^	70 ± 14	859 ± 77	929 ± 77
Vitamin D (mcg/d)	2.45	3.79 ± 0.11 ^b^	2.48 ± 0.17	6.26 ± 0.19 ^b^	0.55 ± 0.11	2.59 ± 0.23	3.14 ± 0.24
Vitamin E (mg/d)	6.53	6.47 ± 0.2 ^b^	6.67 ± 0.34	13.14 ± 0.33 ^b^	1.03 ± 0.2	5.95 ± 0.36	6.98 ± 0.37
Thiamin (mg/d)	1.37	0.84 ± 0.03 ^b^	1.44 ± 0.04	2.28 ± 0.05 ^b^	0.13 ± 0.03	1.41 ± 0.05	1.54 ± 0.05
Riboflavin (mg/d)	1.71	0.86 ± 0.03 ^b^	1.82 ± 0.05	2.68 ± 0.06 ^b^	0.13 ± 0.03	1.81 ± 0.06	1.94 ± 0.06
Niacin (mg/d)	16.28	5.3 ± 0.2 ^b^	16.4 ± 0.5	21.7 ± 0.5 ^b^	1.1 ± 0.3	17.0 ± 0.06	18.0 ± 0.6
Potential niacin (mg/day)	13.2	5.4 ± 0.3 ^b^	13.8 ± 0.4	19.2 ± 0.4 ^b^	1.1 ± 0.3	13.8 ± 0.4	14.9 ± 0.4
Vitamin B6 (mg/d)	1.62	0.91 ± 0.03 ^b^	1.63 ± 0.04	2.54 ± 0.05 ^b^	0.17 ± 0.03	1.67 ± 0.05	1.84 ± 0.05
VitaminB12 (mcg/d)	4.65	1.61 ± 0.05 ^b^	5.15 ± 0.28	6.77 ± 0.28	0.25 ± 0.05	5.72 ± 0.31	5.96 ± 0.31
Folate (mcg/d)	230	138 ± 4 ^b^	246 ± 7	384 ± 8 ^b^	18 ± 4	236 ± 7	255 ± 8
Pantothenic acid (mg/d)	4.93	2.77 ± 0.08 ^b^	5.23 ± 0.13	7.99 ± 0.15 ^b^	0.36 ± 0.08	5.11 ± 0.17	5.47 ± 0.19
Biotin (mcg/d)	36.2	20.6 ± 0.5 ^b^	37.5 ± 1.1	58.1 ± 1.2 ^b^	2.6 ± 0.6	37.1 ±1.3	39.7 ± 1.4
Vitamin C (mg/d)	68.1	51.7 ± 1.4 ^b^	73.3 ± 3.5	124.9 ± 3.7 ^b^	6.7 ± 1.4	71.3 ± 3.8	77.9 ± 4.0

^†^ Comparison of the ONS + DA group with the DA group for each type of intake (ONS only, Diet only, and Diet + ONS). The intake from the diet plus ONS corresponds to the total intake (Diet + ONS), but occasionally very small discrepancies exist due to rounding errors affected by the number of decimal points used. The alphabetic symbols indicate the significance values ^a^ <0.01, ^b^ <0.001. The results are adjusted for age, sex, Charleston Comorbidity Index, MUST category and baseline intake values. DA: Dietary Advice; ONS: oral nutritional supplements.

## Data Availability

The original contributions presented in this study are included in the article. The data supporting the conclusions of this article will be made available by the authors on request for ethical reasons. Further inquiries can be directed to the corresponding author.
